# Hering Hermeneutics: Supplement to *Translation and Commentary of Hering* (1899) by Strasburger et al.

**DOI:** 10.1177/2041669518815921

**Published:** 2018-12-06

**Authors:** Gerald Westheimer

**Affiliations:** Division of Neurobiology, University of California, Berkeley, CA, USA

**Keywords:** Ewald Hering, vernier acuity, hyperacuity, eye movements, neural processing

## Abstract

Strasburger et al.’s welcome translation of Hering’s seminal paper, and reminder of what Hering actually said about eye movements and spatial averaging in vernier acuity, is supplemented by references to further trends on how the subject has evolved to the present state of knowledge.

A place on anyone’s short list of the most creative vision scientists would certainly be found for Ewald Hering. The last of his significant contributions appeared in 1899, ringing down the curtain on a remarkable century of fundamental contributions to vision science. It concerned the gap between the apparent limit of visual acuity imposed by the size of the retinal receptors and the much lower values that had been reported for vernier and stereo targets. Hering’s proposition, widely read as explaining low vernier thresholds by an averaging of local signs along contours, was taught throughout the first half of the 20th century but, because it ran counter Ludvigh's 1953 to claim for good alignment acuity for just two dots, became less of an accepted dogma than a heuristic starting point for more searching work in the second half of the 20th century.

As our discipline rolls into the 21st century, Hering’s (1899) report to the Royal Saxonian Academy is beginning to assume a yet different cast. Science now plays a role in society much like religion did in centuries past. And, like in religion, revealed texts are subjected to textual analysis: The meaning of words is agonized over, attempts are made to fathom the original intent and prevailing knowledge at the time of writing, and adherence to the primary source and alternative interpretations are explored. Hering’s writings often provide something akin to a revelation, and it is, therefore, entirely appropriate that his 1899 paper on the limits of visual acuity should undergo hermeneutic analysis. In the present universally Anglophone environment, this has to start with a translation into English, performed expertly and thoughtfully by Strasburger, Huber, and Rose (2018). These authors also include quotations from and citations of a wide selection of the representative literature. When Helmholtz wrote about the topic in the early 1860s, he had no reason to believe that there was a problem, but subsequent experimental work, especially that of A.W. Volkmann, had suggested very low, subreceptor dimension thresholds. By the mid-1890s, as the German optical industry began to bring in reliable measurements, no doubt was left that in the contest between simplistic viewing of resolution as limited by receptor lattice grain and actual human psychophysical thresholds, the latter had won.

The basic concepts involved in Hering’s proposition are not under dispute, then or now. In the human fovea, the receptor mosaic is not far from hexagonal, each element has its own separate, ordered, indivisible spatial label (Raumwert in Hering, spatial value in Strasburger, significantly not “local sign” in either), and light from even a point extends over several receptors. But Hering went one step further—what counts for purposes of assigning location is only whether or not the receptor responds, not the magnitude of excitation. In other words, for Hering, the anatomical light-accepting receptor grain, indisputably made up of individual discretely spaced modules, has its counterpart in an equally discretely spaced lattice of perceptual spatial values, each signaling its occupancy in only an “all-or-none” fashion. This is not necessarily denied by an empirical approach involving psychometric curves, where the observers’ responses are restricted to two categories, say, “right” or “left.” The low, subreceptor dimension thresholds are derived from the statistical analysis of data runs of many qualitative responses and say nothing quantitatively about the perceptual offset of a just detectably misaligned vernier pattern.

Still, he needed to explain how such small position shifts could manage to generate a net localization difference in an ensemble of relative large elements each being constrained to a yes or no contribution to perception. Here, Hering brings in eye movements. If in a single presentation the pattern’s misalignment happens to be lost in the coarseness of the spatial value grain of receptors, it would reemerge on subsequent “transitory but recurring” (“vorübergehende, aber sich wiederholende Merklichkeit,” in Strasburger et al.’s translation “temporary but repetitive”) detections in subsequent eye displacements. Thus, to achieve superior (with respect to receptor lattice spacing) relative position information about two target features, several samples would be needed and these would be acquired on sequential replacements of the image caused by even small movements of the eye. Contrary to what we so widely imputed to him, Strasburger et al. remind us, Hering never explicitly proposed spatial averaging along the line’s length for purposes of fine localization. Yet, to be candid, neither did he contradict it. In a footnote, he is quite unequivocal about an averaging process: Because of incessant eye movements “… the relative spatial values of the individual line elements fluctuate within certain small limits around a central value, which is the decisive factor in perception.” But it would seem that he was referring to the line’s cross-section rather than its length. Still, support for the widely accepted notion that Hering meant that the whole border and not only a short segment is used might be seen when he says that the border line’s “apparent position … is determined by the space values (breadth values) of all the visual field elements on which the image of the line falls.”

Thus, careful perusal of Hering’s text in the context of Strasburger et al.’s exegetical disquisition encourages their originalist view that, contrary to widely expressed opinion, averaging of local signs along vernier contours was not a contention promoted by Hering, who favored a mechanism in which small eye displacements would shift target patterns around different parts of the coarse retinal mosaic.

Hering’s way of inclusion of eye position change in the apparatus for fine spatial localization is still far from the eye movement emphasis given in later theories. True, he does mention “incessant small eye movements … constantly shifting the image,” but these he regard as relevant to explaining how a line looks smooth and straight and not serrated, as it would were location of the activated retinal elements its immediate basis.

Hering’s view can be counterposed to that in an exactly contemporaneous paper (Stratton, 1900), detailing how that author had (a) convinced himself by a test conducted at a viewing distance of 120 m that alignment thresholds were indeed a few seconds of arc, (b) recognized that the receptor mosaic was much coarser, and (c) understood that light would always be spread over several receptors. Stratton makes explicit that, even under the best circumstances, the thinnest line stimulus would still excite several adjoining receptors, albeit to a varying degree. But unlike Hering, he thought that the receptor *output* would be graded: two adjacent receptors could receive *and signal* quantitatively different excitation levels. Thus, Hering and Stratton, using basically the same experimental findings and the same model of optical spread and retinal anatomical structure, postulated very different retinal output patterns when a vernier stimulus was just detectably misaligned. Hering, medical-trained professor of physiology (albeit more open to arguments involving perception than almost any physiologist of his time), was imbued with the all-or-none nature of nerve conduction and felt that this would apply to retinal receptor output. Stratton, psychologist, Wundt-trained PhD, struggling to break loose psychology from the philosophy department at Berkeley as Wundt had done in Leipzig, felt no constraint about the universality and inevitability of the then prevailing all-or-none doctrine and was willing to allow receptor output to be graded. It took another couple of decades before it would be understood that receptor signals could indeed give rise to quantitative information through the frequency of all-or-none impulses, and a further half-century before the complex analog multistage retinal circuitry from receptors to ganglion cell would be charted.

Stratton’ theorizing on how a fine position value might arise from a much coarser, albeit differentially responding, arrays of receptors was vague:

… the exact seat of the exciting cause … be determined with a degree of accuracy depending on the fineness of discrimination for intensive differences and for catching their interrelation, rather than upon the number and distance apart of the several terminals.

His *interrelation* is presumably not too far from Hering’s “Mittelwerth,” both tentative phrasings which would soon enough blossom into the now accepted averaging mechanism. Stratton, however, did not require displacements to sample different placements on the receptor mosaic.

When the topic was next taken up by Weymouth^1^ and collaborators in the 1920s, the two factors came to the fore. Fixational instabilility had become accepted and, because of its relevant magnitude and time course, had to be folded into the fabric of the acuities. Weymouth and coworkers (Anderson & Weymouth, 1923; Averill & Weymouth, 1925) sought to factor it out by shortening exposure duration. They then postulated that the output from all the receptors affected by the image of a line, light spread plus movement, would participate in an averaging process, generating a “center of gravity.” The averaging process involved the direction normal to the line’s length, that is, the direction of vernier offset. There will be

… a “center of gravity” of all the points stimulated and hence the local sign to which they give rise. The average or mean of these points, which determines the local sign of the straight line, is therefore not restricted to such units as interconal distance or cone diameter but may be accurate to a small fraction of these units just as the mean of a number of measurements made in inches may be accurate to a small fraction of an inch. (Anderson & Weymouth, 1923, p. 586)

Eye movements were looked at from a different perspective by Marshall and Talbot (1942). Like Hering, they regarded them as beneficial, specifically that in sweeping the image over the receptors they generated a temporal gradient rather than sequential relocations. That is, rather than eye movements providing different samples of the retinal mosaic, for Marshall and Talbot, what was important was the time derivative in any given location. Marshall and Talbot’s positive take on micronystagmus was, however, not universally shared (Ratliff, 1952), leading to image stabilization experiments, which came to occupy the attention of the vision community for two decades.

The challenge to our understanding of the mechanism by which fine spatial localization transcends the grain of the neural elements, to which Hering’s insight and authority alerted the field in 1899, continues. Anderson and Weymouth’s procedure, state of the art at the time, is described in great detail and left them to conclude that when the exposure duration is reduced from 1,500 milliseconds to 30 milliseconds, vernier thresholds rise by a factor of about two—implying that the added instability of the eye in the longer exposure duration aided vernier performance. With the improved technology available 50 years later and with the facility of equating light flux across duration, something Weymouth et al. could accomplish only partially, Westheimer and McKee (1977) found vernier thresholds substantially unchanged when exposures were reduced to 11 milliseconds. Whatever role eye movements may play, they are not necessary. Instead recourse seems to have to be taken to an averaging process, long imputed to Hering if perhaps, as suggested by Strasburger et al. with insufficient justification, but clearly enunciated by Anderson and Weymouth, and endowed with renewed experimental support in the demonstration (Westheimer & McKee, 1977) that relative spatial location has hyperacuity values for any light pattern laid down in a 2 to 3 arcminute zone during a 100 to 200 milliseconds epoch.

Tracing the history of the topic illustrates the productive interplay between the study of the whole organism’s behavior and that of its biological structure and function. One would never discover hyperacuity thresholds just from anatomy; conversely, it took Hering’s genius to realize how the apparent limitations of the relative coarse receptor apparatus can be transcended by subtle neural processing.

## Note

1 I got to know Frank Weymouth 40 years later when, to supplement his meager retirement package from the Stanford Physiology Department, he was associated with the U.C. Berkeley Optometry School. In his late 70 s, he was a kind, gentle, socially conscious colleague with a rich knowledge and experience in vision research. It should be mentioned that the impetus to his (as well as Hering’s, Bourdon’s [1902], and Stratton’s) interest in spatial localization came from the realization that stereoacuity had values by far exceeding ordinary visual acuity, and their forays into vernier were predicated by the conceptional simplification of restricting consideration to one retina.
